# Synthetic Biology Strategies for the Development of Live Attenuated Influenza Viruses: Recent Advances and Applications

**DOI:** 10.3390/v18070715

**Published:** 2026-06-29

**Authors:** Kai Yang, Guangtao Yang, Yunxin Xia, Xia Ou

**Affiliations:** School of Basic Medical Sciences, Kunming University of Science and Technology, Kunming 650500, China; 17785630832@163.com (K.Y.); 15808605395@163.com (G.Y.); 15284933990@139.com (Y.X.)

**Keywords:** influenza virus, synthetic biology, attenuated, cancer immunotherapy, vaccine

## Abstract

Influenza viruses, due to their simple genomic structure and potent immunostimulatory capacity, have been extensively explored for applications in cancer immunotherapy and viral vector vaccine development. However, wild-type influenza viruses possess inherent risks of lethal pathogenicity and transmissibility, which limit their direct application. Special cold-adapted influenza strains have been widely used in live attenuated vaccines, which rely on specific amino acid mutations. With the advancement in synthetic biology and reverse genetics technologies, a variety of next-generation attenuated influenza virus have been developed, including genome-recoded viruses, miRNA-targeted viruses, viruses containing premature termination codons, and proteolysis-targeting recombinantviruses. This study systematically summarized the synthetic biology-based strategies for generating a next-generation method for the attenuated influenza virus, critically discussed the advantages and limitations of each strategy, and further analyzed their applications and challenges in cancer therapy and viral vector vaccine development. By synthesizing current research progress, this review aimed to provide a theoretical basis for constructing safer, more stable, and more controllable influenza virus engineering platforms, and to offer new insights for the design of attenuated influenza virus suitable for tumor therapy and novel vaccine delivery.

## 1. Introduction

Influenza viruses belong to the family *Orthomyxoviridae*. They are enveloped, segmented, negative-sense single-stranded RNA viruses with a genome composed of eight independent segments, which encode 17 essential and non-structural proteins for influenza viruses A and B. They contain seven segments for influenza viruses C and D. Because the hemagglutinin (HA) and neuraminidase (NA) genes are prone to antigenic drift and antigenic shift, influenza viruses can cause seasonal epidemics every year and may even trigger global pandemics, posing a persistent threat to public health. In response to this challenge, the development of safe and effective vaccines has become central to influenza prevention and control. In the 1960s, scientists successfully screened cold-adapted attenuated influenza virus with cold-adapted and temperature-sensitive phenotypes by serially passaging influenza viruses in embryonated chicken eggs or primary chicken kidney cells (PCKCs) under gradually decreasing temperatures. Representative strains include A/Ann Arbor/6/60, B/Ann Arbor/1/66, A/Leningrad/134/17/57, and B/USSR/60/69 [1,2,3]. These attenuated strains replicate efficiently only in the nasal cavity, whereas their replication in the lower respiratory tract is restricted. Following intranasal inoculation, they can mimic the natural infection process of influenza viruses and induce humoral immunity, mucosal immunity, and cytotoxic T-cell responses against viral antigens, thereby providing effective protection against influenza. Herein, A/Leningrad/134/17/57 also possesses three major phenotypes: attenuation (att), temperature sensitivity (ts) and cold adaptation (ca). Together with Ann Arbor/6/60, they represent two classic cold-adapted master donor viruses (MDVs) developed via parallel and independent selection. The two strains share identical phenotypes but differ slightly in their genetic backgrounds.

Notably, the attenuated phenotype of cold-adapted strains can be stably inherited. By co-infecting embryonated chicken eggs with a cold-adapted attenuated strain and a circulating influenza strain, or by using reverse genetics, reassortant viruses can be generated that retain the attenuated phenotype while acquiring the immunogenicity of the circulating strain, thereby providing effective protection against antigenically variant viruses. In 2003, the U.S. Food and Drug Administration (FDA) first approved the intranasal live attenuated influenza vaccine (LAIV), named FluMist^®^, Ultravac^®^, Fluenz^®^ and Nasovac-S^®^, a trivalent LAIV developed using this technology, which was later expanded into a quadrivalent vaccine covering additional subtypes [4,5]. At present, such live attenuated influenza vaccines (LAIVs) remain important tools for the prevention and control of seasonal influenza and influenza pandemics.

Furthermore, the backbone of attenuated influenza virus could be used in more research fields. In a transgenic mouse model that spontaneously develops non-small-cell lung cancer, low-pathogenicity influenza A virus efficiently and rapidly eliminated more than 70% of the initial tumor burden [6]. A recent study using influenza A virus to infect murine melanoma demonstrated that the virus could induce immunogenic cell death in tumor cells and activate antitumor immune responses, thereby significantly inhibiting tumor growth [7]. Recombinant influenza viruses generated by reverse genetics have also been successfully used to deliver antigen genes from different pathogens and to induce corresponding protective immunity [8,9]. In addition, Kawaoka et al. [10] found that introducing specific amino acid mutations into genes encoding the viral polymerase complex enhanced the stability of foreign genes within the viral genome, supporting the application of influenza viruses in gene therapy and next-generation vaccine development. Collectively, these studies highlight the potential of attenuated influenza virus as multifunctional vectors. Among the existing influenza attenuation strategies, cold-adapted attenuated strains are the most widely used. However, this strategy has significant limitations: the attenuated phenotype relies solely on specific amino acid substitutions in a limited number of genes, such as polymerase basic protein 1 (PB1), polymerase basic protein 2 (PB2), nucleoprotein (NP), polymerase acidic protein (PA), or matrix protein (M) [11,12], which poses substantial safety concerns for their application as oncolytic viruses. Moreover, clinical guidelines have clearly prohibited the use of LAIVs delivered via nasal spray in cancer patients and immunocompromised individuals. This indicates that cold-adapted attenuated strains cannot meet the safety requirements for use as oncolytic viruses.

Given the intrinsic limitations of conventional attenuation strategies and the increasing demand for viral stability in emerging applications, researchers have developed multiple strategies for attenuating influenza viruses to overcome these challenges. This review focuses on synthetic strategies for generating attenuated strains using influenza A virus as a vector. We compare the attenuation mechanisms, advantages, and limitations of different approaches and summarize recent progress in the applications of attenuated influenza strains. By doing so, this review aims to provide insights into the design of attenuated influenza virus vectors for vaccine development, cancer therapy, and related fields.

## 2. Novel Development Strategies for Live Attenuated Influenza Viruses

**Figure 1:**
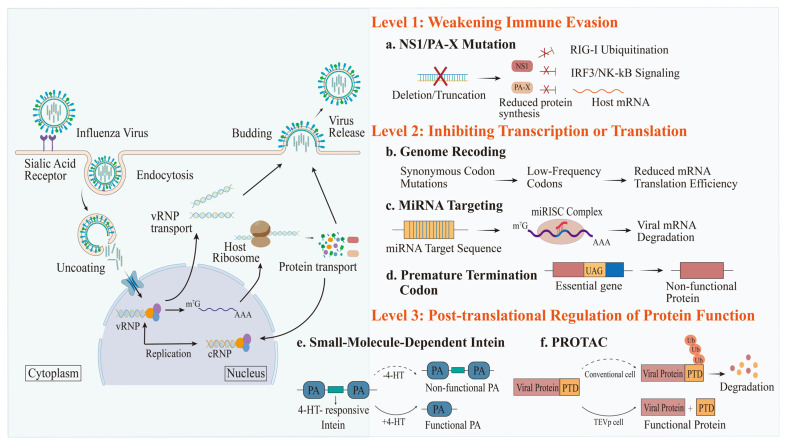
**Influenza virus life cycle and strategies for developing attenuated influenza viruses.** The main steps of viral entry into host cells include attachment via hemagglutinin (HA) to sialic acid receptors, endocytosis into acidic endosomes triggering HA conformational change and release of viral ribonucleoproteins (vRNPs), nuclear entry of vRNPs and transcription/replication by the viral RNA polymerase complex, translation of viral mRNA by host ribosomes, post-translational regulation or modification of viral proteins to achieve functionality, and assembly and budding of progeny virions. The strategies for generating attenuated influenza viruses can be categorized into three levels: (1) attenuation achieved by non-structural protein 1 (NS1)/polymerase acidic X (PA-X) protein mutation or deletion, abrogating viral suppression of the host immune system; (2) inhibition at the transcriptional or translational level, mediated by genome-recoding to restrict mRNA translation efficiency, miRNA targeting to induce viral mRNA degradation, or the introduction of premature termination codons to limit protein synthesis; and (3) post-translational regulation of viral protein activity and stability, accomplished either by protein activation via small-molecule-dependent inteins or by targeted protein degradation using proteolysis-targeting chimera technology.

Based on key stages of the influenza virus life cycle, including replication, transcription, translation, and host–virus interactions, multiple strategies have been developed for generating live attenuated influenza viruses. These include non-structural protein 1 (NS1) or polymerase acidic X protein (PA-X) mutant viruses, genome-recoded viruses, miRNA-targeted viruses, viruses carrying premature termination codons, small-molecule-dependent viruses, and proteolysis-targeting recombinant viruses (Figure 1). The following sections describe each attenuation strategy in detail.

### 2.1. NS1 or PA-X Mutant Viruses

During influenza virus infection, the virus encodes multiple proteins that suppress host gene expression to attenuate antiviral responses, while simultaneously enabling viral mRNAs to preferentially utilize the host translation machinery, thereby coordinately promoting viral replication and immune evasion. For example, NS1 binds to tripartite motif protein 25 (TRIM25), thereby inhibiting the ubiquitination of RIG-I and blocking IRF3/NF-κB signaling. This interferes with the transcription and expression of interferon (IFN) and interferon-stimulated genes (ISGs), ultimately impairing the host’s antiviral capacity [13,14]. PA-X, which possesses endonuclease activity, targets and degrades host mRNAs transcribed by RNA polymerase II, suppressing host protein synthesis and thereby facilitating viral replication [15,16]. Based on these mechanisms, researchers have proposed strategies that involve modifying the coding regions of NS1 or PA-X to attenuate the virus’s ability to antagonize interferon responses or suppress host gene expression, thus generating live attenuated influenza viruses.

Previous studies have shown that various influenza viruses with NS1 deletions or truncations exhibit significantly attenuated replication capacity and pathogenicity in normal cells or mice [17,18,19]. Recently, Hancková et al. [20] used a reverse genetics system to construct a recombinant influenza virus expressing an NS1 protein lacking both the effector domain and the C-terminal domain. In Vero and IC-21 cells, the titer of this virus was substantially lower than that of the wild-type A/WSN/33 strain. Gao et al. [21] demonstrated that an H9N2 virus with PA-X deletion exhibited diminished suppression of host protein synthesis, leading to reduced replication capacity and pathogenicity in mice. It is known that PA-X and NS1 do not function independently to suppress host gene expression [16]; instead, they act synergistically. PA-X cleaves host mRNAs via its endonuclease activity, which dissociates poly(A) binding protein cytoplasmic 1 (PABPC1) from mRNAs and exposes its nuclear localization signal. Meanwhile, NS1 sequesters poly(A) binding protein nuclear 1 (PABPN1), thereby eliminating the competitive inhibition of PABPN1 on the binding sites of nuclear polyadenylated RNAs. Through the synergistic regulation of PABPC1 nuclear translocation and PABPN1 sequestration, PA-X and NS1 shut down host gene expression and prioritize viral gene expression. This serves as a core strategy for the efficient replication of influenza viruses. These combined effects promote the nuclear accumulation of PABPC1, which may contribute to the nuclear retention of viral mRNAs or their preferential engagement with viral transcripts, thereby facilitating viral gene expression.

Avanthay et al. [22] constructed recombinant influenza viruses with NS1 deletion, PA-X deletion, or combined NS1/PA-X deletion based on the pH1N1/09 strain. Their study showed that the NS1/PA-X double-mutant recombinant virus exhibited a 100-fold reduction in viral titer in MDCK cells compared with the wild-type virus and was the only recombinant virus that was significantly attenuated in T3 porcine bronchial epithelial cells. It has been confirmed that introducing PA-X mutations into NS1-truncated viruses can potentiate their attenuation and improve the safety of embryonated chicken egg inoculation. Combined modification of NS1 and PA-X greatly weakens the ability of the virus to antagonize host immune responses, leading to a remarkable reduction in viral replication capacity and pathogenicity [23]. Given that influenza virus strains differ substantially in the basal activity of PA-X and NS1 as well as their functional interaction patterns, mutations will disrupt the original functional homeostasis of the virus. Host environments and the regulatory effects of other viral genes further drive phenotypic divergence of the mutants. Accordingly, PA-X and NS1 mutations exhibit distinct phenotypes across different viral strains [24,25]. For this reason, a universal modification strategy is not applicable to these two proteins, and mutation combinations need to be customized according to the characteristics of individual strains. Furthermore, excessive attenuation may compromise viral immunogenicity, and the low replication efficiency in embryonated chicken eggs or MDCK cells poses a constraint on large-scale production. Future efforts should focus on optimizing this strategy for specific strains to achieve an appropriate balance between attenuation and immunogenicity.

### 2.2. Genome-Recoding Viruses

Owing to the degeneracy of the genetic code, the same amino acid can be encoded by multiple synonymous codons. However, organisms exhibit codon usage bias, a phenomenon in which certain codons are used at frequencies far exceeding those statistically expected. A similar bias exists for codon pair usage [26,27,28,29,30]. Accordingly, researchers have proposed codon or codon pair deoptimization strategies, in which hundreds to thousands of synonymous nucleotide mutations are introduced in a targeted manner into the influenza virus genome without altering the amino acid sequences of the encoded viral proteins. By increasing the number of infrequently used codons or underrepresented codon pairs, these viruses exhibit markedly reduced translation efficiency and protein expression levels in host cells, thereby suppressing viral replication.

Multiple studies have confirmed that recombinant influenza viruses carrying recoded PB1, nucleoprotein (NP), HA, or NA genes display attenuated replication in mice [31,32,33,34,35]. For example, Fan et al. [32] avoided the packaging signals and alternatively spliced regions located at the gene termini and introduced 351 synonymous codon mutations across the eight gene segments of A/Brisbane/59/2007, generating the recombinant virus BR59-8-mut. Compared with the wild-type virus, BR59-8-mut exhibited a 100-fold reduction in replication capacity in A549 cells. Dong et al. [36] replaced approximately 62% of the NA gene of an influenza A virus with synonymous codons to construct a recombinant virus. This recombinant virus showed a dramatically reduced viral titer in mouse lungs while eliciting robust humoral, cellular, and mucosal immune responses. Beyond modulating codon usage frequency, another recoding strategy focuses on altering dinucleotide abundance. The CpG/UpA dinucleotide enrichment strategy increases the abundance of CpG and UpA dinucleotides in the influenza virus genome, thereby activating the zinc-finger antiviral protein (ZAP)-mediated innate immune pathway to suppress viral replication [37,38,39]. Sharp et al. [39] introduced 126 CpG sites into the PB2 gene to construct a recombinant virus enriched in CpG dinucleotides. Their study demonstrated that the attenuation of this virus was dependent on ZAP recognition: viral titers in normal A549 cells were reduced by approximately 100-fold, whereas replication capacity was restored in ZAP-deficient cells. Animal experiments showed that this virus was highly attenuated in mice, and a single low-dose inoculation induced potent humoral and cellular immune responses.

In summary, the genome-recoding strategy introduces a large number of synonymous nucleotide mutations into the viral genome, thereby preserving the native antigen while greatly reducing the likelihood of virulence reversion through mutation. Although this strategy offers significant advantages in terms of in vivo attenuation and immunogenicity, some recoded viruses are severely restricted in replication within current vaccine production platforms (e.g., embryonated chicken eggs, MDCK cells), making large-scale production difficult. In the future, efforts should focus on developing compatible production platforms (such as ZAP-knockout cell lines). However, for current mechanistic studies, ZAP-knockout cell lines do not need to fully meet vaccine manufacturing qualifications. If such cell lines are to be used for formal vaccine evaluation in the future, we will conduct complete qualification and validation in accordance with relevant guidelines to meet equivalent regulatory requirements.

### 2.3. miRNA-Targeted Viruses

MicroRNAs (miRNAs) are a class of non-coding RNAs approximately 18–22 nucleotides in length that broadly participate in regulating critical physiological processes such as cell differentiation, proliferation, immune responses, and stress responses [40]. During influenza virus infection, host miRNAs can interfere with viral replication either by directly targeting viral genes or by modulating host immune-signaling pathways, representing an important mechanism of the host antiviral response [41,42]. The underlying modes of action can be divided into two main categories [40,43]: one involves complete base-pairing with target mRNA, leading to mRNA degradation; the other involves incomplete base-pairing with the 3′ untranslated region (3′UTR) of target mRNAs, primarily repressing translation. Notably, miRNA expression exhibits species-specificity, meaning that certain miRNAs are expressed only in particular species. Taking advantage of this property, researchers have proposed inserting miRNA target sequences into key genes of influenza viruses to allow host miRNAs to modulate viral protein expression, thereby achieving regulated control of viral replication.

MicroRNA-93 (miR-93) is broadly expressed in humans and mice but is absent in embryonated chicken eggs. One study [44] inserted miR-93 target sequences into the NP gene of A/Puerto Rico/8/34 and successfully generated a genetically stable attenuated influenza virus. The 50% lethal dose (LD_50_) of this recombinant virus in mice was 4.1 × 10^5^ PFU, which was 100-fold higher than that of the wild-type virus, demonstrating a markedly attenuated phenotype. Gao et al. [45] inserted several tandemly repeated miRNA-192 target sequences downstream of the stop codon of the NP gene and found that recombinant viruses harboring six or nine copies exhibited attenuated properties, with LD_50_ values ten times higher than those of the wild-type virus. Notably, another study [46] reported a recombinant influenza virus engineered to express an exogenous miRNA that targets and represses the host genes, Cdc2-like kinase 1 (CLK1) or SON DNA-binding protein (SON), thereby restricting viral replication. The results showed that infection with this recombinant virus significantly reduced both mRNA and protein expression levels of CLK1 or SON in A549 cells. Moreover, when mice were infected with a lethal dose of this virus, no severe body weight loss or mortality was observed.

These studies indicate that miRNA-based attenuation strategies effectively reduce influenza virus pathogenicity by inhibiting viral or host genes, providing a reliable approach for the generation of attenuated influenza virus strains. However, the expression of miRNAs is environment-dependent, and alterations in miRNA expression profiles in diseased populations may lead to the restoration of virulence in miRNA-targeted viruses. Additionally, miRNAs carry off-target risks that may cause unpredictable cytotoxicity. To overcome these limitations, future optimization can be pursued in three aspects: (1) screening highly specific miRNAs and designing fully complementary miRNA target sequences to reduce off-target effects; (2) employing multi-target miRNA combinations or combining them with other attenuation strategies to synergistically enhance attenuation and prevent mutation risks; and (3) developing miRNA-knockout cell lines to enable efficient large-scale production of recombinant viruses. Currently, these gene-edited cell lines are used only for basic mechanistic studies, not for vaccine production or potency testing, and therefore do not require vaccine validation.

### 2.4. Premature Termination Codon Viruses

Premature termination codon (PTC)-containing viruses are generated by introducing stop codons (e.g., UAG, UGA) into essential viral genes, causing premature termination of viral mRNA translation in host cells. This leads to the loss of functional proteins and the inability to complete the viral replication cycle, thereby drastically reducing viral pathogenicity [47,48,49]. To address the challenge of efficient production of PTC-containing viruses, researchers [50] established a transgenic cell line stably expressing the *Methanosarcina barkeri* pyrrolysyl-tRNA synthetase (MbPylRS) and tRNA CUA, based on the orthogonal translation system of *M. barkeri*. Through the coordinated action of MbPylRS, tRNA CUA, and an unnatural amino acid (UAA), this cell line is able to read through the introduced stop codons in viral mRNAs and incorporate the UAA, thereby producing full-length functional viral proteins and enabling efficient in vitro production of PTC viruses. Si et al. [48] employed genetic code expansion technology to introduce a large number of amber stop codons (UAG) into the PA, PB2, PB1, and NP genes of the influenza A virus strain A/WSN/33, successfully constructing the replication-defective influenza virus PTC-4A. Mice inoculated with PTC-4A at a dose of 10^9^ PFU—LD_50_ of a wild-type virus being 8 × 10^3^ PFU—showed no significant body weight loss or mortality, and viral titers were virtually undetectable in the lungs. Notably, co-infection with this recombinant virus and wild-type virus produces progeny viruses containing amber stop codons, thereby substantially reducing the risk of genetic reassortment.

Recently, a study [51] reported that anticodon-engineered tRNA (ACE-tRNA) technology enables efficient in vitro production of PTC viruses. This approach modifies the tRNA anticodon to allow endogenous aminoacyl-tRNA synthetases to recognize the introduced stop codons, eliminating the requirement for an exogenous synthetase. Compared with genetic code expansion technology, ACE-tRNA technology markedly improves the readthrough efficiency of engineered stop codons. At the same time, it exhibits extremely low readthrough efficiency at natural stop codons, thereby minimizing adverse effects on the virus and the host cell. However, PTC sites are genetically unstable and are particularly prone to reversion mutations in RNA viruses with high mutation rates. Future efforts should integrate bioinformatics approaches to predict PTC sites with high readthrough efficiency and conservation, and to establish engineered cell lines stably expressing ACE-tRNAs. This would lay the foundation for a platform capable of large-scale production of genetically stable PTC viruses, enabling a rapid response to potential viral transmission.

### 2.5. Small-Molecule-Dependent Viruses

Inteins are polypeptide sequences with self-splicing activity. During post-translational processing, an intein precisely excises itself from the precursor protein and ligates the flanking exteins through a peptide bond, thereby converting an inactive precursor into a functionally mature protein [52,53]. Exploiting this property, Buskirk et al. [54] inserted the human estrogen receptor ligand-binding domain (ER-LBD) into the *Mycobacterium tuberculosis* RecA intein, constructing the first 4-hydroxytamoxifen (4-HT)-dependent intein-based molecular switch capable of conditionally controlling protein function in cells. Subsequently, Peck et al. [55] further optimized the screening system and obtained a second-generation 4-HT-dependent intein, which markedly improved the splicing efficiency for proteins such as GFP, mCherry, and glioma-associated oncogene homolog 1 (Gli1), with splicing activity strictly dependent on the presence of 4-HT. Accordingly, researchers have proposed inserting the gene encoding the 4-HT-dependent intein into key genes essential for influenza virus replication and using 4-HT to regulate viral protein function, thereby generating conditionally replicating live attenuated influenza viruses.

PA, a core subunit of the influenza virus polymerase complex, is essential for viral genome replication and transcription. Chen et al. [56] inserted the above-mentioned 4-HT-dependent intein gene into the coding region of the viral PA gene and successfully constructed a 4-HT-dependent attenuated influenza virus, designated strain S218. At the cellular level, replication of the S218 virus was controlled by 4-HT: in the presence of 4-HT, the viral titer reached 10^4^·^3^ TCID_50_/mL at 72 h post-infection, with replication capacity reduced by approximately 100-fold compared with the wild-type virus; in the absence of 4-HT, no cytopathic effect was observed in virus-infected cells. In vivo experiments in mice demonstrated that this virus was highly attenuated and did not cause pulmonary pathological damage, with its replication in vivo strictly dependent on exogenously supplied 4-HT. More recently, the same group [57] applied the S218 virus to treat melanoma in mice and found that it significantly suppressed tumor growth and lung metastasis, with no marked body weight loss or toxicity observed during the treatment period, further confirming the safety of this virus.

Collectively, the above studies indicate that the attenuation strategy based on small-molecule-dependent intein switches combines several advantages, including operational simplicity, a high safety profile, scalability for manufacturing, and cross-platform versatility. Nevertheless, this technology still faces potential challenges: endogenous estrogen-like substances may activate the intein, leading to off-target viral replication, and the intein itself, as a foreign sequence, carries the risk of eliciting host immune responses. Future optimization efforts will focus on engineering the ER-LBD to reduce its sensitivity to endogenous estrogen-like substances and introducing degron elements to accelerate the clearance of excised free intein fragments, thereby enhancing the safety and controllability of this platform.

### 2.6. Proteolysis-Targeting Recombinant Viruses

Since the Sakamoto group [58] developed the Protac-1 molecule and successfully induced the degradation of methionine aminopeptidase-2 (MetAP-2), proteolysis-targeting chimera (PROTAC) technology has progressively evolved into a novel protein regulation strategy. This technology employs bifunctional molecules that simultaneously bind a target protein and an E3 ubiquitin ligase, promoting target protein ubiquitination and subsequent recognition and degradation by the host ubiquitin–proteasome system, thereby modulating protein expression [59,60]. Inspired by this technology, researchers have proposed inserting proteasome-targeting peptides into the influenza virus genome to promote the ubiquitination of viral proteins, thereby enabling targeted degradation of viral proteins via the host ubiquitin–proteasome system to regulate viral replication.

Si et al. [61] inserted a proteasome-targeting domain (PTD) consisting of a proteasome-targeting peptide (ALAPYIP) and a tobacco etch virus cleavage site (TEVcs)-containing linker (ENLYFQG) upstream of the stop codon of the M1 gene, constructing the attenuated influenza virus M1-PTD. When this virus infects conventional cells, the von Hippel–Lindau (VHL) protein recognizes the proteasome-targeting peptide within the PTD and mediates the ubiquitination of the M1 protein via an E3 ubiquitin ligase, leading to its degradation by the host proteasome and consequent suppression of viral replication. In cells expressing tobacco etch virus protease (TEVp), however, TEVp cleaves the TEVcs, releasing the proteasome-targeting peptide from the M1 protein and thereby restoring viral replication capacity. Compared with the wild-type virus, the M1-PTD virus exhibited a 100-fold reduction in replication capacity in conventional cells and could be stably propagated for more than 20 passages. To overcome the limitation that PTD insertion sites were restricted to the C-terminus of genes, the same group [62] performed a genome-wide screen and identified six novel candidate sites (PA-D294, NS1-L163, PA-N350, M1-M135, M1-H222, and the M1 C-terminus), successfully constructing recombinant attenuated viruses capable of engaging either Von Hippel-Lindau (VHL) or beta-transducin repeat-containing protein (β-TrCP) E3 ubiquitin ligases. Three days after intranasal inoculation, the viral titer of this virus in mouse lung tissue was 10^3^ PFU, approximately 200-fold lower than that of the wild-type virus.

Compared with cold-adapted attenuated strains, PROTAC-based viruses exhibit superior attenuation. However, their clinical application still faces several challenges. For instance, the expression levels of E3 ubiquitin ligases such as VHL and β-TrCP vary among individuals; in particular, individuals with deficient E3 ubiquitin ligase expression or those receiving protease inhibitor therapy may be at risk of attenuation failure. Furthermore, as a foreign peptide, the PTD may act as a neoantigen and carry the risk of inducing an autoimmune response. To address these limitations, future studies may consider introducing multi-PTD cooperative designs and exploring alternative degradation pathways, such as the autophagy–lysosome system, so as to overcome variability in E3 ubiquitin ligase expression and reduce the likelihood of virulence reversion. In addition, bioinformatics-based screening of PTD sequences with low immunogenicity could further enhance the safety and applicability of this platform.

## 3. Application of Live Attenuated Influenza Virus

Table 1 systematically summarizes the research progress on the development of influenza attenuated strains based on different attenuation strategies. With the successful implementation of multiple influenza attenuation strategies, the virulence of attenuated influenza strains has become stable and controllable, substantially improving their safety when used as live attenuated influenza vaccines. This progress signifies that the influenza virus is transitioning from a natural pathogen into a controllable biological tool. Consequently, the application of live attenuated influenza viruses is no longer confined to the development of conventional influenza vaccines but is progressively expanding into multiple directions, including vaccine platforms and oncolytic viruses.

### 3.1. Application as a Viral Vector Platform in Vaccine Development

Currently, more than ten viral vectors—including adenovirus, vesicular stomatitis virus, vaccinia virus, and measles virus—are commonly used in vaccine development. Among these, attenuated influenza virus strains used as viral vectors [65,66] offer distinctive biological advantages: (1) mucosal route immunization effectively activates humoral, mucosal, and cellular immunity, which is particularly important for preventing infection by pathogens that invade via mucosal surfaces; (2) the flexibility to exchange foreign antigen genes enables rapid responses to antigenic variants or emerging pathogens; and (3) the stably inherited attenuated phenotype ensures the safety of vaccination.

In recent years, numerous studies have reported influenza-vectored vaccines targeting COVID-19. The design concept involves inserting antigenic genes, such as conserved T-cell epitopes of SARS-CoV-2 or the spike protein receptor-binding domain (RBD), into the genome of an influenza vector, thereby enabling virus-mediated delivery of foreign genes to activate immune responses against SARS-CoV-2. Chen et al. [67,68] constructed an influenza-vectored vaccine expressing the RBD based on an NS1-deleted A/California/04/2009 backbone. This vaccine effectively activated humoral immunity, mucosal immunity, tissue-resident memory (TRM) T cells in the upper respiratory tract, and trained immunity, significantly ameliorating SARS-CoV-2-variant-induced lung injury in hamsters. Moreover, clinical studies have further demonstrated that this vaccine possesses excellent safety, tolerability, and user experience, and has shown favorable immunoprotective efficacy against the Omicron variant [69,70,71]. Using an matrix protein 2 (M2)-deficient single-replication (M2SR) influenza virus as a vector, Hill-Batorski et al. developed a bivalent vaccine, SARS-CoV-2 M2SR, carrying the RBD. Intranasal inoculation of hamsters with this vaccine caused no adverse reactions while effectively activating immune responses against both influenza and COVID-19. Furthermore, attenuated influenza vectors have been successfully applied in the development of vaccines against a variety of bacteria (e.g., *Helicobacter pylori*, *Brucella* spp., *Mycobacterium tuberculosis*) and viruses (e.g., tick-borne encephalitis virus, respiratory syncytial virus, human immunodeficiency virus), inducing protective immunity against the respective pathogens [72,73,74,75,76,77].

Nevertheless, attenuated influenza viruses used as vaccine vectors still face considerable limitations. Traditional attenuated influenza strains are often over-attenuated, and defective interfering particles (DIPs) are frequently generated during their production [78]. These DIPs carry short defective viral genomes (DVGs) that have lost the ability to encode complete functional proteins. They preferentially compete for key factors such as RNA-dependent RNA polymerase, nucleoproteins, and envelope proteins through short DVGs, and exploit retained packaging signals to outcompete full-length vRNAs for packaging opportunities. This results in a large number of progeny viruses being non-infectious DIPs, thereby reducing the delivery efficiency and immunogenicity of foreign viral antigens. Pre-existing anti-influenza antibodies, which are widespread in the human population, can neutralize influenza viruses, further impairing the potency of vectored vaccines [79]. Additionally, the packaging capacity of the influenza virus genome is limited, and the insertion of relatively large foreign gene fragments can significantly affect viral replication and genetic stability. In response to these challenges, various optimization strategies have been developed. Novel influenza attenuation strategies that incorporate conditional replication control mechanisms enable precise regulation of the degree of attenuation, thereby effectively balancing the safety and immunogenicity of vectored vaccines. Replacing the HA and NA subtypes on the surface of the viral vector can reduce the neutralizing effect of pre-existing antibodies in the population. The use of 2A self-cleaving peptides or internal ribosome entry site (IRES) elements to achieve polycistronic expression of multiple genes, or the modular partitioning of antigens into different gene segments, can overcome the genetic payload limitations of influenza vectors [80,81]. These optimization strategies provide an important foundation for the application of attenuated influenza vectors in next-generation vaccine development.

### 3.2. Application as Oncolytic Viruses in Tumor Therapy

Influenza virus possesses the ability to selectively infect and lyse tumor cells, and its tropism for tumors is closely related to its unique replication advantage in malignant cells. Unlike normal cells, influenza virus preferentially infects tumor cells because tumor cells overexpress sialic acid receptors, including Neu5Gc, to facilitate viral entry, while their antiviral pathways (especially the IFN-signaling pathway) are defective. Furthermore, the tumor microenvironment is favorable for viral replication. Viral infection and replication induce potent oncolytic effects and immunogenic cell death, thereby activating antitumor immunity [6,82]. At present, research on oncolytic influenza viruses remains at the preclinical stage, and their clinical translation faces considerable safety challenges. To address these challenges, scientists have been actively exploring the use of live attenuated influenza viruses as oncolytic agents, aiming to reduce viral pathogenicity while enhancing oncolytic potency.

Kuznetsova et al. [83] introduced the double mutations S342P and R343I into the HA gene of an NS1-truncated influenza virus, replacing the trypsin cleavage site of HA with an elastase cleavage site to further enhance tumor tropism. Compared with the wild-type virus, this recombinant virus, administered intravenously at a dose of 10^8^ TCID_50_/mL, significantly inhibited the growth of melanoma and pancreatic cancer, while no virus was detected in the brain, lungs, or liver. This modification eliminated the natural restriction that viral activation is dependent on the respiratory tract. However, due to the widespread distribution of elastase in tissues, the virus is capable of causing systemic infection and viremia, thereby introducing risks of enhanced virulence and cytokine storms. Furthermore, elastase is highly conserved across species, which weakens the interspecies barrier and increases the risk of zoonotic transmission. Du et al. [84,85] constructed a hyper-interferon-sensitive influenza virus (HIS) by introducing eight interferon-sensitive mutations into the PB2, matrix protein 1 (M1), and NS1 genes. As a genetically engineered strain, HIS features precise genetic modifications, high sensitivity to interferon, restricted replication, potent immune activation, and excellent safety, resulting in attenuated replication, robust immune responses, and no pathogenicity in immunocompetent hosts. Subsequent studies showed that intranasal administration of HIS at a dose of 10^4^ TCID_50_/mL did not cause distal organ damage or mortality in mice. Compared with the wild-type virus, intratumoral injection of HIS significantly suppressed the growth of a mouse lung cancer model resistant to immune checkpoint inhibitors. Chen et al. [57] demonstrated that intratumoral injection or intranasal inoculation of a 4-HT-dependent attenuated influenza virus inhibited the growth of melanoma and lung metastatic foci in mice, and the oncolytic effect could be further enhanced by co-administration of 4-HT. Notably, this group constructed a folic acid (FA)-modified nanostructured lipid carrier (NLC) capable of targeted delivery of 4-HT to tumor cells, thereby specifically activating the virus within the tumor and enhancing antitumor efficacy without damaging normal tissues. Ji et al. [86] introduced premature termination codons (PTCs) into the PB1, PA, NP, and HA genes of the influenza A virus strain A/WSN/33/H1N1 (WSN), generating a PTC virus. The results showed that intranasal inoculation of this virus at a dose of 10^5^ PFU significantly suppressed lung melanoma metastasis in mice without causing body weight loss or lung injury. However, the intrinsic immunostimulatory capacity of influenza virus is relatively weak and insufficient to overcome the immunosuppressive tumor microenvironment. To address this, they inserted an anti-PD-L1 nanobody gene into the PB2 gene, which further enhanced the inhibitory effect on lung metastatic foci in mice and significantly improved the objective response rate in tumor-bearing animals. Similarly, numerous studies have utilized influenza virus as a vector to deliver immunostimulatory genes into the tumor microenvironment, compensating for the limited immune-activating capacity of the virus itself and thereby achieving more potent oncolytic effects [87,88].

In summary, as a novel oncolytic virus, influenza virus—through genetic modification or drug combination—can enhance its tumor tropism and immune-activating capacity, exhibiting excellent oncolytic efficacy and safety in various tumor types, including hepatocellular carcinoma, non-small-cell lung cancer, and melanoma. Nevertheless, this field still faces core challenges, including insufficient immune stimulation, neutralization by pre-existing antibodies, and difficulties associated with intravenous route administration. To overcome the current therapeutic bottlenecks, it is necessary to engineer novel vectors regulated by tumor-specific promoters to improve safety, explore cell-based carriers or inhalation-based delivery to overcome delivery obstacles, and deeply integrate oncolytic influenza viruses with other therapeutic modalities (such as immune checkpoint inhibitors, radiotherapy, and CAR-T cell therapy) to achieve synergistic antitumor efficacy.

## 4. Conclusions

This paper systematically reviews the strategies for constructing attenuated influenza viruses and their application progress in two major directions: vaccine vectors and oncolytic viruses. To overcome the inherent limitations of conventional cold-adapted attenuated strains in terms of genetic stability and safety, researchers have developed a variety of novel influenza attenuation strategies by reducing viral pathogenicity through three approaches: impairing viral immune evasion, suppressing viral transcription or translation, and modulating viral protein function at the post-translational level. Although these attenuation strategies differ in their technical routes, they share a common contradiction: the more thoroughly the influenza virus is attenuated, the weaker its replication in vivo and the lower its yield in production systems, thereby limiting its efficacy as a vaccine or oncolytic agent. In other words, it is difficult to simultaneously balance viral safety, efficacy, and productivity. For instance, conventional cold-adapted strains, which rely on only a small number of amino acid mutations to achieve an attenuated phenotype, are unable to strike an adequate balance among safety, efficacy, and productivity. Genome-recoded viruses, which incorporate hundreds or thousands of synonymous mutations to reduce translational efficiency, greatly enhance safety but at the cost of reduced production titers and diminished viral efficacy.

Nevertheless, these novel influenza attenuation strategies provide a basis for resolving the trade-off among these three factors. For example, small-molecule-dependent intein-based and proteasome-targeting attenuation strategies couple viral replication capacity to external signals such as 4-HT or TEVp. Supplying the signal during production maintains viral yield, while withdrawing the signal after inoculation renders the virus incompetent, thereby decoupling safety from productivity. miRNA-targeted attenuation strategies link the condition for viral attenuation to host species specificity: high expression of specific miRNAs in normal cells restricts viral replication, whereas the absence of such miRNAs in tumor cells permits efficient viral replication, thereby achieving a separation of safety and efficacy. Thus, the key to balancing these three factors lies not in finding a static optimal compromise, but in designing them so that they no longer constrain one another, allowing each to reach its optimum. The studies by Chen et al. [56,57] further validate this concept: this group constructed a 4-HT-dependent attenuated influenza virus that achieves large-scale in vitro production upon the addition of 4-HT in embryonated chicken eggs or MDCK cells. For in vivo application, they developed a folate receptor (FR)-modified NLC capable of specifically delivering 4-HT to tumor cells, thereby precisely activating the virus within the tumor. This strategy prevents safety, efficacy, and manufacturability from constraining one another—it not only enables efficient in vitro production of the virus but also demonstrates remarkable oncolytic efficacy and safety in melanoma and lung metastasis models. Therefore, future design of attenuated influenza viruses should shift from single attenuation strategies toward multi-layered safety control, combining PTC, codon deoptimization, miRNA targeting, PROTAC-mediated degradation, and other strategies to construct viruses with multiple replication restrictions, thereby reducing the risk of virulence reversion and aberrant replication. Concurrently, engineered complementary cell lines and active targeting strategies should be developed to address the issues of insufficient yield and low efficacy of highly attenuated viruses.

Furthermore, attenuated influenza viruses still face several limitations in their applications. Pre-existing immunity may neutralize the viral vector, impairing in vivo delivery efficiency; certain strategies that rely on exogenous signals may increase production costs and process complexity; oncolytic virus monotherapy exhibits limited efficacy, and intratumoral injection restricts its clinical applicability; intravenous or inhalation delivery faces challenges such as neutralizing antibodies and insufficient targeting. To address these issues, future research should develop viral vectors with rare serotypes or substituted surface proteins to evade pre-existing immunity, and optimize low-cost inducers and production systems. In addition, emphasis should be placed on developing conditional replication elements triggered by tumor hypoxia or aberrant signaling pathways, so as to confine efficient viral replication to lesion sites, while exploring combination therapies with immune checkpoint inhibitors and other modalities to overcome the limitations of monotherapy. Only through simultaneous breakthroughs in both design concepts and application technologies can attenuated influenza viruses truly be realized as a controllable biological platform.

On the other hand, in practice, the research and development of oncolytic viruses inevitably faces multiple technical and regulatory challenges. Despite these hurdles, constructing oncolytic viral vectors based on influenza virus is technically achievable. Given its promising prospects for tumor treatment, relevant regulatory bodies support exploratory research in this area, and candidate products developed from this strategy qualify for application as Class 1 New Drugs. In terms of the quality control and supervision of genetically modified virus seeds, the Center for Drug Evaluation (CDE) has explicitly stipulated strict requirements on the maximum passage number of oncolytic viruses. Specifically, whole-genome sequencing is mandatory for viruses at each passage generation during serial cultivation, so as to guarantee consistent genetic stability and sustained therapeutic potency throughout passaging. Considering that the vector is derived from live influenza virus, there exists a theoretical risk of genetic reassortment between the engineered virus and wild-type influenza strains. Nevertheless, our experimental results confirm that this safety risk is negligible. Since key virulence genes of the oncolytic influenza virus have been genetically modified, the likelihood of spontaneous genetic reassortment has been substantially minimized.

## Figures and Tables

**Table 1:** Summary of attenuated influenza virus strains based on different attenuation strategies.

Attenuation Strategy	Parental Strain	Gene Modification Methods	Infection Model	Attenuated Effect		Reference
NS1 or PA-X mutant	HA and NA derived from the A/Anhui/1/2013 (H7N9), six internal genes derived from A/Leningrad/17	NS1 truncation (NS1-126)	Mouse		Mutated strain is completely nonpathogenic	Prokopenko et al., 2023 [63]
	HA, NA and M derived from the A/Vietnam/1203/04 (H5N1), five internal genes derived from IVR-116	NS1-ORF deletion	Human		No serious adverse events were observed	Nicolodi et al., 2019 [64]
	A/Hamburg/4/2009pdm09(H1N1)	NS1 truncation (NS1-126), PA-X null	Cell (MDCK)	Parental strain	Viral titer of mutated strain is 100 times lower than parental strain	Avanthay et al., 2023 [22]
				Mutated strain		
	NS1-truncated A/turkey/Oregon/71-delNS1 (H7N3) variant	PB2-D309N, PA-X null	Egg	Parental strain	ELD_50_/EID_50_ is 1	Ghorbani et al., 2023 [23]
				Mutated strain	ELD_50_/EID_50_ is 0.49	
Genome recoding	A/Brisbane/59/2007 (H1N1)	Codon synonymous mutation modification in eight segments	Cell (A549)	Parental strain	Viral titer of mutated strain is 100 times lower than parental strain	Fan et al., 2015 [32]
				Mutated strain		
	A/Puerto Rico/8/1934 (H1N1)	Codon synonymous mutation modification in PR8-NA gene	Egg	Parental strain	Viral titer of mutated strain is 100 times lower than parental strain	Dong et al., 2023 [36]
				Mutated strain		
	A/Puerto Rico/8/1934 (H1N1)	Synonymous addition of 126 CpGs into PB2 gene	Cell (A549)	Parental strain	Viral titer of mutated strain is 100 times lower than parental strain	Sharp et al., 2023 [39]
				Mutated strain		
Small-Molecule-Dependent	A/Puerto Rico/8/1934 (H1N1)	4-HT-dependent intein	Mouse or cell	Parental strain	Viral titer of mutated strain is 100 times lower than parental strain	Chen et al., 2023 [56]
				Mutated strain		
Premature termination codon viruses	A/WSN/1933 (H1N1)	Addition of stop codon into PA, PB2, PB1, NP gene	Mouse	Parental strain	LD_50_ is 8 × 10^3^ PFU	Si et al., 2016 [48]
				Mutated strain	10^9^ PFU inoculation does not induce any clinical sign	
MicroRNA-targeting	A/Puerto Rico/8/1934 (H1N1)	Insertion of the microRNA-93 target sequence into NP gene	Mouse		LD_50_ of mutated strain is 100 times more than parental strain	Perez et al., 2009 [44]
	A/Puerto Rico/8/1934 (H1N1)	Insertion of the microRNA-192 target sequence into NP gene	Mouse	Parental strain	10^3^ PFU induces 100% mortality	Gao et al., 2020 [45]
				Mutated strain	LD_50_ is 10^4^ PFU	
	A/Puerto Rico/8-KV20/1934 (H1N1)	Insertion of the artificial microRNAs into NA gene	Mouse	Parental strain	MLD_50_ is 500 PFU	Wen et al., 2022 [46]
				Mutated strain	3 × 10^5^ PFU is nonpathogenic	
Protein degradation-targeting chimeric viruses	A/WSN/1933 (H1N1)	Incorporation of PTD into the M1 gene	Cell (MDCK-TEVp)		Compared with parental strain, the mutant virus exhibits a replication capacity in MDCK-TEVp cells that is reduced by more than 100-fold and is non-replicative in conventional cells	Si et al., 2022 [61]
	A/WSN/1933 (H1N1)		Mouse	Parental strain	LD_50_ is 10^3^ TCID_50_	Zhang et al., 2025 [62]
				Mutated strain	10^5^ PFU is nonpathogenic	

Abbreviations: EID_50_, 50% of the chicken embryo infection dose; ELD_50_, 50% of the chicken embryo lethal dose; TCID_50_, 50% Tissue Culture Infectious Dose; PFU, plaque formation unit; LD_50,_ 50% lethal dose; MLD_50_, 50% mouse lethal dose; IVR-116, a high-growth reassortant vaccine backbone strain recommended by the WHO. It possesses the HA and NA genes from A/New Caledonia/20/99 (H1N1), the PB1 gene from A/Texas/1/77 (H3N2), and the remaining genes (PB2, PA, NP, M, NS) from A/Puerto Rico/8/34 (PR8).

## Data Availability

No new data were created or analyzed in this study.
